# Timing is essential: Humoral and cellular responses to SARS-CoV-2 vaccination in a cohort of patients with auto-immune diseases treated with rituximab

**DOI:** 10.1016/j.heliyon.2024.e38043

**Published:** 2024-09-17

**Authors:** Irène Gallais Sérézal, Laurie Spehner, Marie Kroemer, Inès Bourezane, Nadine Meaux-Ruault, Clément Prati, Andréa Pastissier, Juliette Lodovichetti, Pierre Tiberghien, François Aubin

**Affiliations:** a Department of Dermatology, Besançon University Hospital, Besançon, France; b Université de Franche-Comté, EFS, INSERM UMR RIGHT, Besançon, France; c Inserm CIC-1431, CHU Besançon, Besançon, France; d Department of Pharmacy, Besançon University Hospital, Besançon, France; e Department of Internal Medicine, Besançon University Hospital, Besançon, France; f Department of Rheumatology, Besançon University Hospital, Besançon, France; g Etablissement Francais Du Sang, La Plaine-Saint Denis, Saint-Denis, France

## Abstract

Rituximab (RTX), an anti CD20 monoclonal antibody, is now a gold standard treatment for several auto-immune and chronic inflammatory diseases. Receiving RTX exposes patients to more severe infections as vaccinations become virtually inefficient in terms of B cell responses. During the COVID-19 crisis, RTX–exposed patients exhibited more severe forms of the disease, and in some cases, the introduction of RTX was delayed or avoided to protect patients as much as possible against SARS-CoV-2 infections. We retrospectively collected cellular and humoral responses from thirteen patients with dermatological and rheumatological autoimmune diseases who had been vaccinated after receiving RTX. Memory T cells subsets from patients that exposed to RTX showed few differences when compared to a cohort of healthy donors. The IFN**ᵧ** ELISpot assay using SARS-CoV-Prot_S1 showed that eight patients exhibited a positive response that was neither correlated to the time between RTX infusion and the sampling nor to the time between RTX and the vaccination. Conversely, analysis of the SARS-CoV-2 serology showed a clearly lower binding antibody units per mL in case of recent RTX infusion. The safe threshold forconsistently positive serology was to vaccinate at least 300 days after RTX infusion (p = 0.02). Our data illustrate the difficulty in obtaining a satisfactory response to vaccination after RTX treatment within almost a year after the latest infusion, and emphasize the need to better evaluate the risk of relapses in auto-immune diseases before administering RTX in order to maintain RTX only in patients whose medical situation requires it.

## 1 Introduction

The use of the anti CD20 monoclonal antibody for auto-immune diseases has increased, and dermatology is no exception, as rituximab (RTX) is now a gold standard treatment for pemphigus vulgaris [1]. Anti CD20 antibodies deplete circulating B cells, and require the vaccination of patients against relevant infections before treatment-induction. Nevertheless, the COVID19 crisis added a level of complexity, both for patients already undergoing RTX treatment but also in those for whom the introduction of this medication was envisioned. Indeed, receiving RTX exposed patients to more severe forms of the COVID19 [2,3]. In some cases, the introduction of RTX was therefore delayed, or avoided, to give time to perform an efficient vaccination and protect patients as much as possible against SARS-CoV-2 infections. However, it is not clear to the dermatologists what timeframe should be respected between the latest RTX infusion and the next vaccination for it to be efficient. In this study we present a cohort of patients undergoing rituximab treatment in which we evaluated both the cellular and humoral responses to SARSCoV-2 vaccination.

## 2 Material and methods

Patients were recruited in the departments of dermatology, internal medicine and rheumatology between July 2021 and July 2022. Information regarding their latest SARS-CoV-2 vaccination was collected. Blood cells from anonymous healthy donors were collected at the Etablissement Français du Sang (EFS, Besançon, France) as apheresis kit preparations after the signature of informed consent and following EFS guidelines. A total of 20 mL of blood was drawn upon the subsequent visit of the patients at the hospital. Peripheral blood mononuclear cells (PBMCs) from patients were isolated by density centrifugation on Ficoll gradient (Eurobio). PBMCs were cryopreserved in CryoStor (CS10 and CS5) cell preservation media (Sigma-Aldrich) at −196 °C for flow cytometry and ELISpot assay analysis. Then, 3x10^5^ PBMC/well were cultured in anti-human IFNᵧ monoclonal antibody in ELISpot plate with the SARS-CoV-Prot_S1 PepTivator (1 μg of peptide/mL Miltenyi Biotec) in X-Vivo 15 medium (Lonza) for 48 h at 37 °C. The IFNᵧ– specific T cell responses were quantified by ELISpot assay (Diaclone). Cells were cultured with medium alone or Phorbol-12-myristate-13-acetate/Ionomycin (250 ng/mL; 10 μg/mL, Sigma-Aldrich) and used as negative and positive controls, respectively. Each result presented is the mean of the duplicates. Estimation of specific T cell number was expressed as spot-forming cells (SFC)/3x10^5^ PBMCs and calculated after subtracting negative control values (background), using the C.T.L (Cellular technology limited) Immunospot system and Immunospot 5.0 analyser software. Responses were positive when IFNᵧ spot number was ≥10 and ratio 2-fold above background. Serology IgG antibodies to SARS-CoV-S were detected using the anti-RBD SARS-CoV-S IgG assay on Architect I2000SR (Abbott). Samples with a result ≥50 AU/mL (7.04 BAU/mL) were considered positive according to the manufacturer's instructions. Additionally, for flow cytometry staining, PBMCs were washed and stained for 30 min at 4 °C in PBS/0.01 % BSA and 2 mM EDTA with the following Fixable viability Dye (FvD)-eFluor 780 (eBioscience) and antibodies. Samples were directly acquired on a FACS Lyric (BD biosciences) and analyzed with DIVA software. Statistical analyses were performed using GraphPad Prims 6 software. The level of significance was set at p < 0.05 for all tests (∗p ≤ 0.05 and ∗∗p ≤ 0.01). Continous variables were expressed as a median and statistical differences between groups were tested using Mann-Whitney test.

## 3 Results

**Fig. 1:**
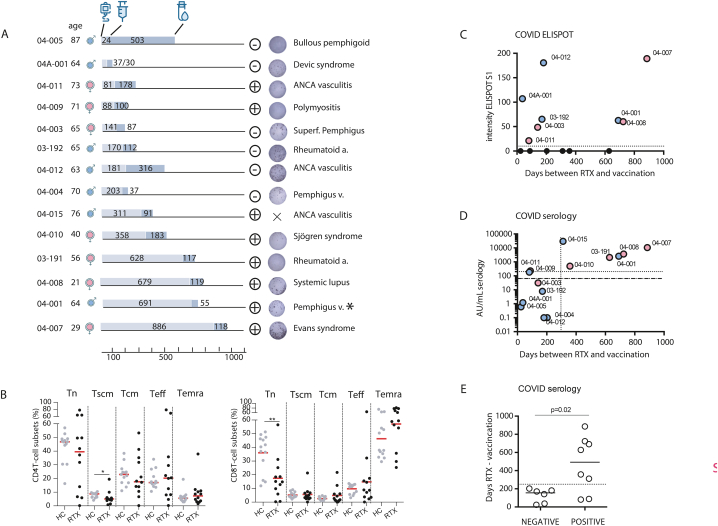
Influence of the time between vaccination and RTX infusion on the humoral and cellular responses to SARS-CoV-2 vaccination A) Schematization of the patients included, the icons represent from left to right: RTX infusion, SARS-CoV-2 vaccination, and blood sampling with the corresponding number of days between each event written in the bars. The±icons refer to the positivity or negativity of the serology using the 50 AU/mL threshold. The ELISPOT images shows the IFN**ᵧ** ELISPOT response to SARS-CoV-2 Spike S1 protein. Controls for the Elispot are shown in Supplementary Fig. 1. Diagnosis are written on the far right: Rheumatoid arthritis, Pemphigus vulgaris, superficial Pemphigus, Evans syndrome (auto-immune hemolytic anemia and thrombocytemia), Systemic Lupus Erythematosus, Polymyositis, Sjögren syndrome, ANCA vasculitis, Devic syndrome (neuromyelitis). B) Memory CD4 and CD8 T cells subsets were analyzed in PBMC of healthy controls (gray, n = 13) and patients (black, n = 12). C-D) Correlation between C) SARS-CoV-Prot_S1 specific immune responses (IFNᵧ spots/3.10^5^ cells, positivity shown as dotted line); or D) SARS-CoV-2 serology level (AU/mL, positivity shown as dashed line), according to the days between the infusion of Rituximab and the vaccination. E) Dot plots of the number of days between the infusion of Rituximab and the vaccination according to the serological responses in patients (negative if the serology was inferior to 50AU/mL). Mann-Whitney test, where ∗p < 0.05 and ∗∗p < 0.01. Abbreviations: RTX: Rituximab, HC: healthy controls, Tn: Naive T cells, Tscm: stem cell-like memory T cells, Tcm: central memory T cells, Teff: effector memory T cells and Temra: Terminally differentiated effector memory T cells. ∗ identifies the patient who had a positive response to protein M in the ELISpot, suggesting a previous infection by SARS-CoV-2. Male and female icons show the gender of patients. Fig. 1C and D: statistical testing with pearson correlations are non-significant.

Fourteen patients were recruited, 6 men and 8 women, age ranged from 21 to 74 years old. One patient received methotrexate simultaneously (03–191), and four received systemic steroids (04–008, 04–009, 04–011, 04–012, 04-A-001), 04–005 had hydrocortisone. The time between the infusion of rituximab and the vaccination differed broadly as shown in Fig. 1A. Vaccination was performed with mRNA vaccination, and triggered both cellular and humoral responses against the spike protein. Here, memory T cells subsets from patients that had been exposed to RTX showed few differences when compared to a cohort of healthy donors (Fig. 1B, T cell populations and gating are presented in Supplementary Fig. 1). The stem cell-like memory T cells were slightly decreased in the CD4^+^ T cell subsets in the patients compared to the healthy controls (8.8 vs 5.1 %, p = 0.013), while naive CD8^+^ T cells were decreased (14.95 vs 40.40 %, p = 0.008, Fig. 1B).

Next, we performed an IFN**ᵧ** ELISpot assay using SARS-CoV-Prot_S1. Eight patients had a positive response that was neither correlated to the time between RTX infusion and the sampling nor to the time between RTX and the vaccination (Fig. 1C and not shown). Of note, one patient had spontaneous T cell responses against SARS-CoV-M protein (#04-001) and none against the SARS-CoV-N (Fig. 1A and not shown). Thus, in our cohort, the level of cellular protection against SARS-CoV-2 was not related by the time since the last RTX infusion. Conversely, analysis of the SARS-CoV-2 serology showed a clearly lower binding antibody units per mL (BAU/mL) in case of recent RTX infusion (Fig. 1D–E). Patients who had received RTX less than 200 days before could for 2/8 have a positive serology, but with a low titer. Conversely, the safe threshold to have a consistently positive serology was to vaccinate at least 300 days after RTX infusion (Mann-Whitney test in Fig. 1E, p = 0.02).

## 4 Discussion

Our data demonstrate that in order to ensure an efficient vaccination, a delay of more than 300 days after the latest RTX infusion is warranted. In auto-immune diseases such as auto-immune bullous diseases, RTX is used as a sustainment therapy to avoid relapses. However, a long-term use of RTX exposes patients to infections, directly or through the impossibility for the vaccination to build an appropriate immune response. Although cellular responses are still ongoing under RTX treatment –as an elegant study recently described [4], the lack of a satisfying titer of antibody does not allow for an optimal defense [5]. According to our findings, the patients should be vaccinated at least 300 days after the previous infusion, and others [6] have suggested to wait until B-cell count reaches again 40/μL before vaccination in RTX–treated patients. However, this strategy is also likely to postpone the planned bi-annual infusions that are recommended in some auto-immune diseases [7]. Antibody titers can be used to optimize the frequency of RTX infusions in some diseases as pemphigus vulgaris [8], but there is a need of better biomarkers or clinical indicators of relapses to shorten the sustainment RTX therapy when it is not necessary anymore in auto-immune diseases.

How long after the latest RTX infusion would a vaccine trigger efficient antibody titers is still an open question: previous work showed that patients still had a suboptimal humoral response to SARS-CoV-2 vaccination even one and a half years after RTX [9]. More work emphasized that one year after RTX therapy, the vaccinal responses were still not optimal [10]. Our data are thus reassuring, possibly because the patients included had been vaccinated at least 2 times against SARS-CoV-2 before their inclusion, as recommended by the national guidelines in France at this time.

Few data are available on the naïve CD8^+^ T cells after vaccination. In a recent study [4], naïve CD8^+^ T cells were found increased in patients undergoing RTX treatment compared to patients with B cell deficiency from another origin, and correlated to a subsequent spike protein–specific CD8^+^ T cell responses after vaccination. We could not perform the same comparison and any direct comparison with their dataset is arduous.

Shortcomings of our study include the limited number of subjects and the retrospective “real-life” setting, in that times between RTX infusion, SARS-CoV-2 vaccination, and sampling were different for each patient. However, this variability allowed for the detection of a clear time-effect between the positivity of the SARS-CoV-2 serology and the period between RTX infusion and the vaccination. Also, no detailed information was available regarding our control population of healthy blood donors, thus no pairing with the patients according to their age or their vaccination status could be performed, as this information could not be retrieved. It would also be of interest to evaluate the impact of the humoral or T cell response induced by a previous SARS-CoV-2 infection before RTX administration, or just before vaccination. In our series, only one patient (#04-001) demonstrated a positive response to protein M associated with both cellular and humoral responses against the spike protein after vaccination.

In conclusion, our data illustrate the difficulty to obtain a satisfying response to vaccination after treatment with RTX within almost a year after the latest infusion, and emphasize the need to better evaluate the risk of relapses in auto-immune diseases in order to sustain RTX only in patients whose medical situation requires it.

## Ethics statement

The COV-CREM study (Identifier: NCT04365322) was carried out in accordance with GCP-ICH-6 and conducted in a single university-affiliated hospital. Eligible patients were screened during hospital stay or medical consultation and received all information relating to the study. Oral informed consent was obtained by investigators before inclusion.

## Data availability statement

All data will be made available upon request.

## CRediT authorship contribution statement

**Irène Gallais Sérézal:** Writing – original draft, Visualization, Software, Formal analysis. **Laurie Spehner:** Formal analysis, Data curation. **Marie Kroemer:** Supervision, Resources, Methodology. **Inès Bourezane:** Data curation. **Nadine Meaux-Ruault:** Writing – review & editing, Data curation. **Clément Prati:** Writing – review & editing, Data curation. **Andréa Pastissier:** Writing – review & editing, Data curation. **Juliette Lodovichetti:** Writing – review & editing, Data curation. **Pierre Tiberghien:** Methodology, Conceptualization. **François Aubin:** Writing – review & editing, Data curation, Conceptualization.

## Declaration of competing interest

The authors have no conflict of interest to declare, please see statement in the manuscript.
