# Supplementary figures and images for: Timing is essential: Humoral and cellular responses to SARS-CoV-2 vaccination in a cohort of patients with auto-immune diseases treated with rituximab
Source: Heliyon. 2024 Sep 17;10(18):e38043. doi: 10.1016/j.heliyon.2024.e38043 (PMC11425176; doi:10.1016/j.heliyon.2024.e38043)

A

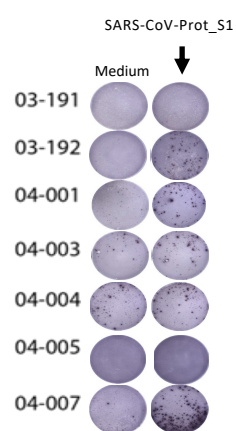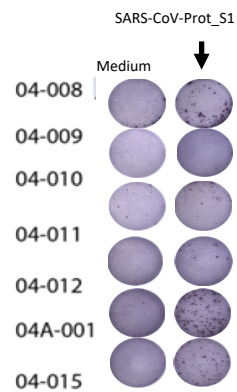

B

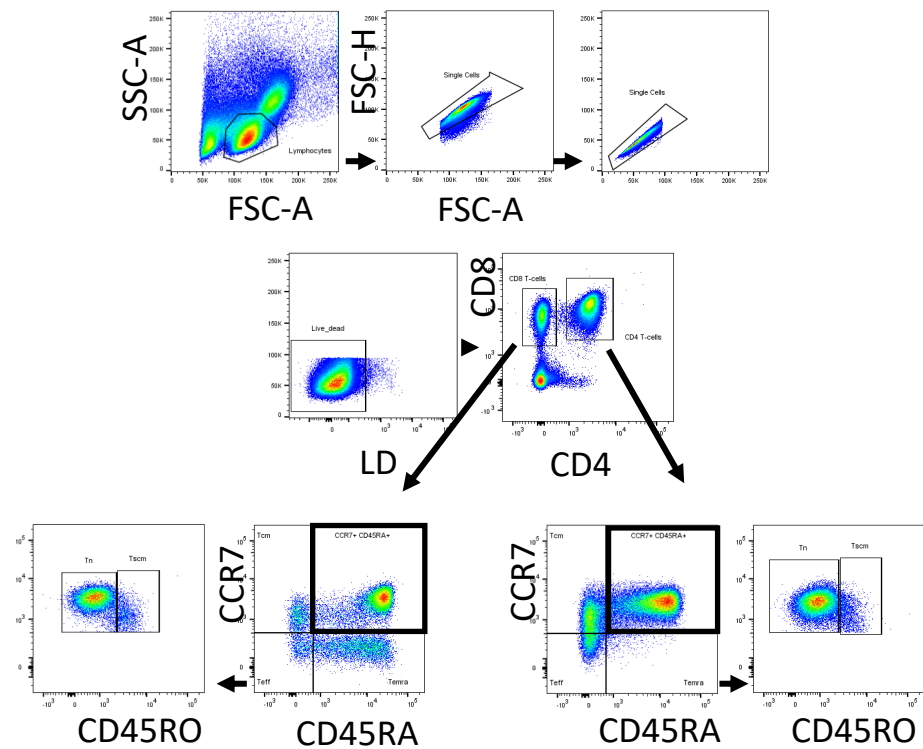

Supplement: Multimedia component 1 [file mmc1.pdf]
